# Atypical Delayed-Onset Endophthalmitis Following Intravitreal Dexamethasone Implant Managed Without Implant Removal: A Rare Case Report and Literature Review

**DOI:** 10.22336/rjo.2024.64

**Published:** 2024

**Authors:** Amit Nandan Tripathi, Vipin Rana, Sandepan Bandopadhyay, Jaya Kaushik, Pradeep Kumar

**Affiliations:** 1 Department of Ophthalmology, Command Hospital, (Eastern Command), Kolkata, India; 2 Department of Ophthalmology, Command Hospital, (Central Command), Lucknow, India; 3 Department of Ophthalmology, Army Hospital, (Research & Referral), New Delhi, India

**Keywords:** Dexamethasone implant, endophthalmitis, intravitreal injection, Ozurdex, DEX = Dexamethasone, CRVO = Central retinal vein occlusion, BCVA = Best-corrected visual acuity, HMCF = Hand movements close to face, PPV = Pars plana vitrectomy, RVO = Retinal vein occlusion, Anti-VEGF = Anti-vascular endothelial growth factor, IOP = Intraocular pressure, IVABs = Intravitreal antibiotics, PCR = Polymerase chain reaction

## Abstract

**Objective:**

To report a case of atypical delayed-onset endophthalmitis following intravitreal dexamethasone (DEX) implantation, managed successfully without implant removal.

**Case presentation:**

A 72-year-old Asian woman with recurrent macular edema due to central retinal vein occlusion (CRVO) received an intravitreal DEX implant. Two weeks post-injection, she experienced blurred vision but no pain or redness. Best-corrected visual acuity (BCVA) had dropped to hand movements near the face (HMCF). Examination revealed 3+ anterior chamber cells and a 1.5 mm hypopyon, with significant vitreous haze obscuring retinal details. A diagnosis of acute endophthalmitis was made. Initial treatment with intravitreal vancomycin and ceftazidime was followed by pars plana vitrectomy (PPV) without implant removal. Microbiological tests were negative, and vision improved significantly, with BCVA returning to 6/12 after two weeks.

**Discussion:**

Endophthalmitis following DEX implantation is rare, and its management is not well-defined. While implant removal is often recommended, favorable outcomes can be achieved without it. The negative culture results and atypical presentation suggested a possible non-infectious etiology. Intraocular steroids may obscure typical signs of infection.

**Conclusion:**

Atypical delayed-onset endophthalmitis following DEX implantation can be successfully treated with prompt vitrectomy and intravitreal antibiotics without implant removal, underscoring the need for individualized management in such cases.

## Introduction

Intravitreal dexamethasone implants (DEX; Ozurdex, Allergan, Inc., Irvine, CA) are an established therapy for managing macular edema associated with diabetes, retinal vein occlusions (RVO), and posterior uveitis. The safety and efficacy of DEX implants have been confirmed in large randomized trials, such as the GENEVA and MEAD studies [1,2]. DEX implants offer sustained therapeutic benefits, potentially reducing the need for frequent anti-vascular endothelial growth factor (anti-VEGF) injections, especially in macular edema cases resistant to these therapies.

Although complications from DEX implants are rare, typical side effects include cataract formation in phakic patients and elevated intraocular pressure (IOP) [3]. Endophthalmitis, a serious but infrequent complication, occurs more commonly with steroid injections than with anti-VEGF treatments. Steroid injections increase the risk of endophthalmitis by 6.92 times (0.13% vs. 0.019%), possibly due to the larger bore needle used for steroids (27 or 25 G for triamcinolone and 22 G for DEX, compared to 30 or 32 G for anti-VEGF agents) and the immunosuppressive effects of steroids, which lower the threshold for bacterial infection [4]. Endophthalmitis rates following DEX implantation range from 0% to 1.3%, with no cases reported in the GENEVA study and a 1.3% rate in the HURON study.

Only a few cases of post-injection endophthalmitis following DEX implant have been reported in the literature [5-13]. Most were treated with implant removal. To our knowledge, only two cases successfully managed without implant removal have been reported [11,13], both presented with typical endophthalmitis symptoms. We present a unique case of acute endophthalmitis following an intravitreal DEX implant in a 72-year-old woman, notable for its atypical presentation and successful treatment with 25-gauge vitrectomy and IVABs without implant removal.

## Case report

A 72-year-old Asian woman with no history of hypertension or diabetes presented with recurrent macular edema in her right eye, secondary to central retinal vein occlusion (CRVO). Despite multiple anti-VEGF injections, macular edema recurred. Her best corrected visual acuity (BCVA) was 6/12 before her latest treatment. After informed consent, she received an intravitreal DEX implant under sterile conditions in the operating theatre, with moxifloxacin 0.5% eye drops administered before and after the procedure. The patient was discharged with a 5-day regimen of topical moxifloxacin.

**Fig. 1:**
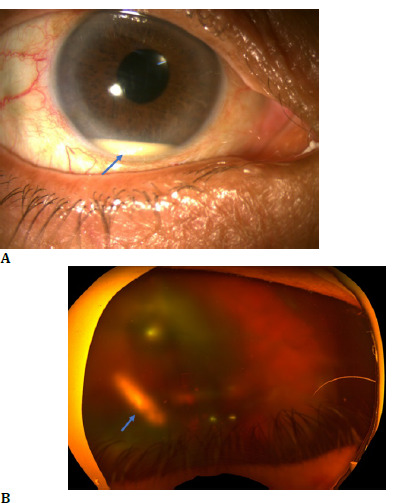
Right eye (**A**) anterior segment showing hypopyon on day of presentation (blue arrow); (**B**) ultra-wide fundus photo showing vitreous haze with media clarity grade 4 on day of presentation with Ozurdex implant in situ (blue arrow)

**Fig. 2:**
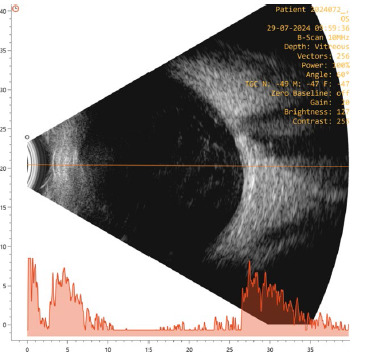
Right eye AB-scan ultrasonography showing dot-like echoes in the vitreous indicative of inflammation, with an attached retina on the day of presentation

Two weeks post-injection, the patient reported blurred vision for three days but denied pain, redness, or photophobia. Examination showed her BCVA had dropped to hand movements close to the face (HMCF), with an intraocular pressure (IOP) of 18 mm Hg. The anterior segment showed 3+ anterior chamber cells and a 1.5 mm hypopyon (**Fig. 1 A**), with no corneal haze or circumcorneal congestion. The vitreous was hazy, obscuring the retina (**Fig. 1 B**). AB-scan ultrasonography revealed increased intravitreal echoes indicative of inflammation, with an attached retina (**Fig. 2**). A diagnosis of acute post-DEX implant endophthalmitis was made.

The patient received intravitreal vancomycin (1 mg/0.1 ml) and ceftazidime (2.25 mg/0.1 ml) after a vitreous tap for culture and polymerase chain reaction (PCR) analysis on the same day of presentation. After 24 hours with no improvement, she underwent 25-gauge pars plana vitrectomy (PPV) and anterior chamber wash. Although implant removal was initially planned, it was abandoned as the implant could not be cut with a 25-gauge vitrectomy cutter. Intravitreal vancomycin, ceftazidime, and amphotericin B (5 µg/0.1 ml) were administered post-vitrectomy.

**Fig. 3:**
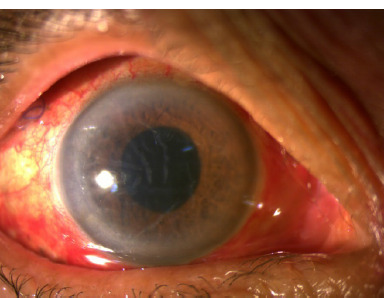
Right eye anterior segment showing no hypopyon on POD 1

**Fig. 4:**
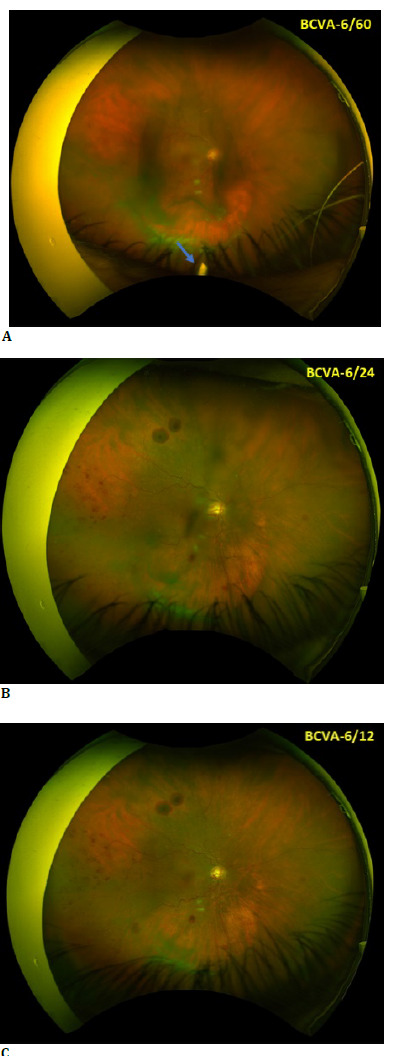
Right eye fundus photo. (**A**) POD 1 showing Ozurdex implant (blue arrow); (**B**) POD 7 and (**C**) POD 14

**Fig. 5:**
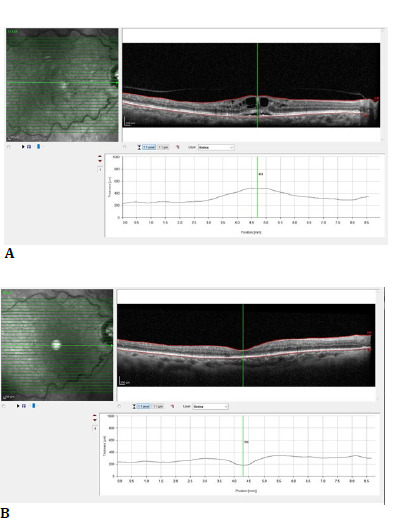
Right eye optical coherence tomography (**A**) before Ozurdex implant showing cystoid macular edema; (**B**) two weeks after Ozurdex implant

Microbiological cultures and viral PCR were negative. The patient’s vision rapidly improved, with no hypopyon by postoperative day (POD) 1 (**Fig. 3**), reduced vitreous haze, and BCVA improvement to 6/60 on POD 1 and 6/24 by POD 7. Two weeks later, her BCVA had returned to 6/12 (**Fig. 4 A-C**) with no macular edema (**Fig. 5 B**).

## Discussion

Intravitreal sustained-release DEX implants are effective for managing macular edema secondary to various retinal conditions, with endophthalmitis being an exceedingly rare complication (0.07% to 0.3% of cases) [2]. However, no clear guidelines exist for managing DEX implant-related endophthalmitis, with only a few case reports in the literature (**Table 1**).

**Table 1:** Summary of characteristics and treatment outcomes of endophthalmitis following DEX injection

Study	Year	No. of cases	Onset in days	Vision presentation	Organism Isolated	Treatment	DEX Removal	PPV Done in days	Final Vision
Marchino et al. [5]	2013	01	02	PL	Alliococcus otitidis	IVAB PPV	YES	07	CF
Yorgun et al. [6]	2014	01	02	HM	Negative	PPV+IVAB	YES	01	6/7.5
Esen et al. [7]	2015	02	03	HM	Negative	IVAB	NO	_	6/60
			03	HM	Negative	IVAB	NO	_	6/36
Mahalingam et al. [8]	2017	01	03	PL	CONS (NOT TYPED)	PPV+IVAB	YES	01	6/36
Goel et al. [9]	2017	01	15	CF	CONS (NOT TYPED)	IVAB PPV	YES	02	6/18
Stem et al. [10]	2017	02	NA	6/240	Staphylococcus epidermidis	IVAB	NO	_	6/15
			NA	HM	Staphylococcus epidermidis	PPV	YES	NA	6/240
Bastakis GG et al. [11]	2018	01	04	6/120	Staphylococcus epidermidis	PPV+IVAB	NO	01	6/19
Salceanu et al. [12]	2019	01	04	CF	Staphylococcus lugdunensis	IVAB PPV	YES	01	6/36
Al Zamil W et al. [13]	2019	01	03	CF	Negative	IVAB PPV	NO	30	6/60
Chin YYB et al. [14]	2024	03	07	6/30	Negative	PPV+IVAB	YES	60	6/75
			35	6/50	NA	Topical therapy	NA	NA	6/30
			15	CF	NA	Topical therapy	NA	NA	6/37.5

DEX = Dexamethasone implant, PPV = Pars Plana Vitrectomy, IVAB = Intravitreal antibiotics, NA = Not Available, CONS = Coagulase-negative Staphylococcus, HM = Hand Movement, CF = Counting Fingers, PL = Perception of Light

Some clinicians advocate for implant removal during vitrectomy, as the implant may act as a reservoir for infection or hinder the immune response. Favorable outcomes have been reported with implant removal during PPV in several cases [5,6,8,10,12,14]. However, removal can be technically challenging due to the implant’s material properties [9]. In contrast, other cases, including those by Bastakis et al. [11] and Al Zamil et al. [13], achieved good visual outcomes with PPV without implant removal. In our case, the patient regained pre-infection visual acuity. Additionally, two cases were successfully managed with IVABs alone, without the need for PPV or implant removal [7,10].

The unique feature of our case was delayed presentation with white eyes and only hypopyon. The patient had no ocular complaints typical of endophthalmitis. Our literature review conducted via PubMed, Embase, and Scopus found only three similar cases of atypical presentation reported by YYB Chin et al. [14]. Two were treated with topical steroids and antibiotics alone, while one required PPV and implant removal due to persistent inflammation. Notably, cultures in several cases [6,7,13], including ours, were negative, suggesting a non-infectious etiology. Intraocular steroids may have masked typical signs of infection.

So far, only one study has reported non-infectious (sterile) endophthalmitis post intravitreal Ozurdex but did not share any further details of their findings [15].

While sterile endophthalmitis has been reported with intravitreal triamcinolone, it is rarely seen with DEX implants. In this case, the atypical symptoms and delayed onset were consistent with sterile endophthalmitis, although a definitive cause could not be identified.

Intraocular contamination often originates from pathogens on the lid margin and conjunctiva. Evidence suggests that povidone-iodine application is as practical as topical antibiotics in reducing bacterial load [16]. Aerosolized droplet contamination from the oropharyngeal tract has also been identified as a risk factor for endophthalmitis [17]. Preventative measures are essential, such as wearing masks and minimizing talking during procedures. The larger 22-gauge needle used for DEX implants may increase contamination risk, requiring careful scleral injection technique [5].

## Conclusion

Atypical delayed-onset post-intravitreal DEX endophthalmitis, characterized by painless, white eyes, is rare. Although all cases should initially be treated as infectious, our case showed that prompt pars plana vitrectomy and intravitreal antibiotics could yield favorable outcomes without implant removal.
